# Surge in Fentanyl Toxicity Deaths in Jefferson Parish, LA, 2013-2018

**DOI:** 10.31486/toj.19.0039

**Published:** 2019

**Authors:** Kayla Noto, Dana Troxclair, Melva Williams, Ronda Conners, Erin Mathes, Mark Bone, Gerry Cvitanovich

**Affiliations:** ^1^ Loyola University New Orleans, New Orleans, LA; ^2^ Department of Forensic Pathology, Jefferson Parish Coroner's Office, Harvey, LA; ^3^ Department of Toxicology, Jefferson Parish Coroner's Office, Harvey, LA; ^4^ Department of Death Investigation, Jefferson Parish Coroner's Office, Harvey, LA; ^5^ Coroner, Jefferson Parish Coroner's Office, Harvey, LA

**Keywords:** *Analgesics–opioid*, *death*, *drug overdose*, *fentanyl*

## Abstract

**Background:** The opioid epidemic in the United States has resulted in a significant increase in fentanyl-related overdoses nationwide since 2013. Because of an increased rate of fentanyl-related overdoses seen in Jefferson Parish, LA, the Jefferson Parish Coroner's Office (JPCO) hypothesized that the opioid epidemic, specifically related to fentanyl, had reached this region. To test this hypothesis, JPCO analyzed fentanyl overdose deaths to determine if the deaths had increased during a 6-year period and if the change met the definition of an epidemic.

**Methods:** In the toxicology laboratory at JPCO, enzyme-linked immunosorbent assay screening and gas chromatography/mass spectrometry are used in-house to determine the presence of drugs. Drug-positive samples are sent to National Medical Services (NMS) Labs to quantify the analyte concentration in each sample. Data for each decedent were extracted from the Medicolegal Death Investigation Log (MDILog) database for the years 2013 through 2018.

**Results:** A slow increase in fentanyl-related deaths during the first 3 years of the study period was followed by a near doubling of cases in 2016, a tripling of cases in 2017, and a 6-fold increase in incidence in 2018. During the 6-year study period, fentanyl-related deaths increased from 6 in 2013, to 8 in 2014, to 14 in 2015. Twenty-five deaths in 2016 spiked to 66 in 2017 and increased to 78 fentanyl-related overdose deaths in 2018. The percentage of fentanyl-related overdose deaths increased from 4% of all drug deaths in 2013 to 45% in 2018.

**Conclusion:** The data validate the hypothesis that the fentanyl epidemic has affected Jefferson Parish in southeast Louisiana.

## INTRODUCTION

Fentanyl, a synthetic opioid analgesic drug, is 50 to 100 times more potent than morphine.^1^ This fast-acting drug metabolizes into norfentanyl, hydroxyfentanyl, hydroxynorfentanyl, and despropionylfentanyl which are clinically inactive and irrelevant.^2^ As a pharmaceutical, fentanyl is commonly used to manage postoperative pain and treat severe cancer-related pain that is no longer manageable with traditional opioid treatment.^1^ Fentanyl, similar to oxycodone and hydrocodone, which are more commonly prescribed, has become a commonly abused drug. When used recreationally, fentanyl, fentanyl analogs, and opiates may lead to respiratory depression, seizures, muscle rigidity, hypotension, coma, and death.^3^ The illicit synthesis of fentanyl has led to an increase in fentanyl analogs, structurally different compounds that have essentially the same physiologic effects as the parent drug, fentanyl, on the human body.^3^ Fentanyl analogs include norfentanyl, acetyl fentanyl, furanyl fentanyl, acryl fentanyl, cyclopropyl fentanyl, and methoxyacetyl fentanyl. The appearance of fentanyl analogs in the recreational drug community led to a sudden increase in the number of drug deaths above the number normally expected, thereby meeting the definition of an epidemic.^4^ Prior to 2013, intermittent deaths occurred from fentanyl-laced heroin. Since 2013, the fentanyl epidemic has led to numerous deaths across the country when the drug was combined with heroin or used alone in concentration.^5^

In 2005^6^ and 2007^7^, the US Food and Drug Administration (FDA) published public health advisories warning citizens of the dangers of fentanyl. The FDA specifically advised that fentanyl should not be used for short-term pain or by patients who do not already use narcotic painkillers because of the potency of fentanyl compared to other opioids.^7^ Likewise, in 2013, the Centers for Disease Control and Prevention (CDC) released a health advisory to warn citizens of the dangers of fentanyl and its analogs.^8^ In 2015, the United States Drug Enforcement Administration (DEA) released a nationwide alert on fentanyl as a threat to health and public safety.^9^ In 2018, the DEA released a drug and chemical evaluation of fentanyl, reinforcing that licit use is only intended for the management of cancer-related or persistent/chronic pain in patients who are already receiving opioid medication or who require continuous opioid analgesia.^10^

Reports from across the United States show increases in deaths related to these substances. A study was conducted in Marion County, IN to assess the fatal opioid overdoses in the county from 2010 through 2015.^11^ Of the 1,199 cases identified, 918 involved an opioid drug such as heroin, fentanyl, morphine, or codeine, and 185 (20%) involved the use of fentanyl. From 2010 to 2015, fentanyl-related overdoses in Marion County, IN increased from 15 cases to 63 cases, a 64% rate of annual change. However, during the same time interval, the number of fentanyl prescriptions written in Marion County, IN decreased 8.9%.^11^ In a similar study in the Chicago metropolitan area, the Cook County Medical Examiner's Office reported fentanyl-related deaths each month during 2015.^5^ From January to October, the number of fentanyl-related deaths increased from 2 to more than 25.^5^ In response to the surging numbers of fentanyl-related deaths, in October 2015, the CDC released another health advisory informing citizens of the dangers of fentanyl.^1^

The 2015 CDC health advisory provided data on fentanyl confiscations and overdoses in states across the country. The highest concentrations were seen in the northeastern region of the United States. Ohio and Massachusetts topped the list of total fentanyl confiscations in 2014, accounting for almost 2,000 reports. Other than Florida, no southern or western state made the 2014 top ten list, suggesting that the opioid epidemic had not reached the southern or western parts of the United States.^1^

## METHODS

To test whether the opioid epidemic had reached the southern region of the United States, an autopsy-based study was conducted at the Jefferson Parish Forensic Center in Louisiana to assess and analyze deaths resulting from the use of fentanyl and its analogs for the period 2013 through 2018. Age, race, and sex were used to identify the demographic most affected by the fentanyl death outbreak.

### Investigation and Autopsy

At the Jefferson Parish Coroner's Office (JPCO), autopsies are conducted for all suicides, homicides, and accidents and for any case in which the coroner determines that the death was unexpected. Prior to the autopsy, death investigators gather information at the scene about where and how the death may have occurred. The necessity of an autopsy is determined after the investigation. During the autopsy, samples are taken for toxicology (drug) testing. Blood, urine, and vitreous humor are the 3 primary fluids taken at autopsy for toxicology testing.

### Data Collection

The JPCO uses the Medicolegal Death Investigation Log (MDILog), an internet-based case management system for coroners and medical examiners (Occupational Research and Assessment, Inc.), to store all information collected during death investigations, including scene writeups, photos, autopsy findings, and toxicology results that are used to determine the cause and manner of each death. For this study, data were extracted from the MDILog database for the 6-year span (January 2013 through December 2018) to determine the incidence of fentanyl-related overdose deaths in Jefferson Parish, LA.

In general, cases are considered accidental drug-related overdoses if (1) the forensic pathologist classifies the manner of death as an accident and (2) a drug or drugs are listed as the primary cause of death on the death certificate. For this study, fentanyl-related deaths were defined as any death that occurred as the result of the presence of fentanyl and/or fentanyl analogs alone or in combination with other illicit drugs, alcohol, and/or prescription drugs, and the drug was stated as the cause of death on the death certificate.

### Laboratory Methods

The JPCO Toxicology Laboratory qualitatively screens for major classes of drugs by enzyme-linked immunosorbent assay (ELISA), a competitive binding process that involves antigen-antibody complexes. All positive ELISA results are confirmed by gas chromatography/mass spectrometry (GC/MS) that identifies the drugs (eg, fentanyl analogs) by ion detection. Once the drugs are identified by GC/MS, the samples are sent to National Medical Services (NMS) Labs in Horsham, PA for quantitative analysis. High performance liquid chromatography, time of flight mass spectrometry, and/or liquid chromatography with tandem mass spectrometry are used to quantify the concentration of each drug analyte detected in the decedent's fluoridated blood. The results are recorded in ng/mL. The toxicology results are uploaded to the MDILog database.

We conducted an MDILog search to identify every drug overdose death attributed to fentanyl and/or its analogs during the study period. Data collected were demographics of decedents, number of cases, types of analogs identified, and quantified values of each analyte.

### Statistical Analysis

We calculated the fentanyl-related death frequencies and proportions during the study period and compiled descriptive reports of fentanyl-related deaths and fentanyl analog presence from the toxicology data. Microsoft Office Excel 2010 v.16.9.1 was used for data analysis.

## RESULTS

### Incidence of Drug Overdose Deaths

**Figure 1.**
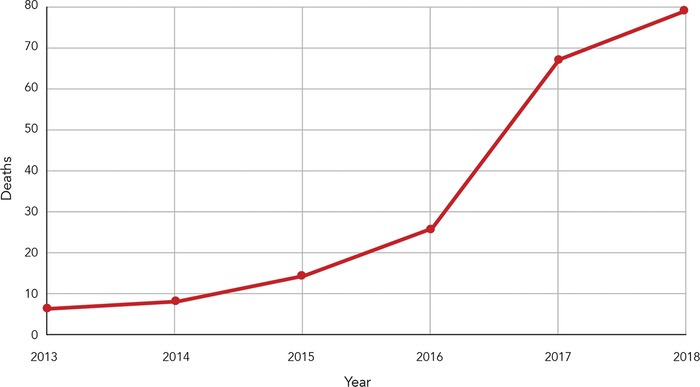
**Fentanyl-related overdoses in Jefferson Parish, LA, 2013-2018.**

In Jefferson Parish, LA, between January 2013 and December 2018, 922 deaths were attributable to drug overdose, an average of 12.8 drug overdoses per month. Of the 922 total drug deaths, 197 (21%) were reported as fentanyl-related deaths. The Table and Figure 1 show that deaths attributed to fentanyl toxicity increased year over year, and by 2018 accounted for 45% of drug-related deaths. These numbers represent an increase from 0.5 cases per month in 2013 to 6.5 cases per month in 2018.

**Table.** Fentanyl-Related Deaths in Jefferson Parish, LA Relative to All Drug Overdose–Related Deaths, 2013-2018

Year	Fentanyl-Related Deaths, n	All Drug Overdose–Related Deaths, n	Percentage of Fentanyl-Related Deaths
2013	6	144	4%
2014	8	131	6%
2015	14	145	10%
2016	25	154	16%
2017	66	175	38%
2018	78	173	45%
Total	197	922	21%

### Description of Fentanyl/Fentanyl Analog Deaths

**Figure 2.**
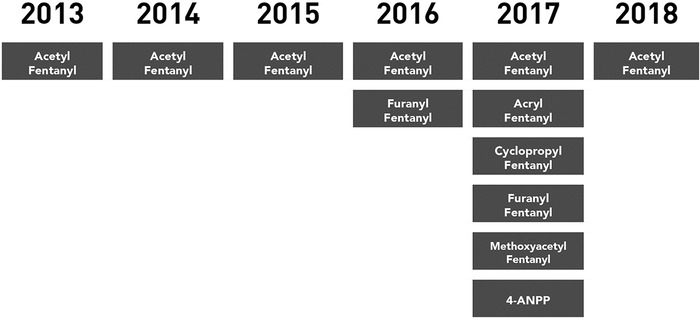
**Fentanyl analogs identified in fentanyl-related deaths in Jefferson Parish, LA, 2013-2018.** 4-ANPP, 4-anilino-N-phenethylpiperidine.

Figure 2 shows analog proliferation by year. In 2013, 2014, and 2015, acetyl fentanyl was the only analog present in the 28 total cases. Among the 25 fentanyl-related deaths in 2016, 2 analogs of fentanyl were identified: acetyl fentanyl and furanyl fentanyl. In 2017, 6 analogs of fentanyl were reported in 66 deaths: acryl fentanyl, acetyl fentanyl, furanyl fentanyl, 4-ANPP (4-anilino-N-phenethylpiperidine), cyclopropyl fentanyl, and methoxyacetyl fentanyl. In 2018, only the acetyl fentanyl analog was detected.

Because 2017 was the most proliferative year for analog detection, we investigated the different combinations of fentanyl analogs that led to overdoses. Seventy-six percent (50 of 66) of cases included the parent drug fentanyl, either alone or in combination with a fentanyl analog. Analogs were present in 47% (31 of 66) of all fentanyl deaths in 2017.

**Figure 3.**
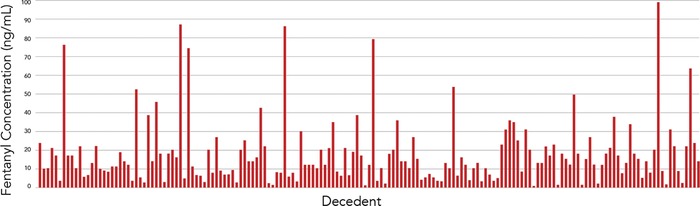
**Postmortem concentrations of fentanyl detected in 165 decedents in Jefferson Parish, LA, 2013-2018.**

Overall, average postmortem concentrations were as follows: the fentanyl concentration was 17.62 ng/mL (range, 0.57-100 ng/mL); norfentanyl was 3.47 ng/mL (range, 0.13-33 ng/mL); acetyl fentanyl was 103 ng/mL (range, 0.1-750 ng/mL); acryl fentanyl was 1.42 ng/mL (range, 0.14-5.8 ng/mL); and furanyl fentanyl was 5.93 ng/mL (range, 0.23-49 ng/mL). Cyclopropyl fentanyl and methoxyacetyl fentanyl were qualitatively detected. Figure 3 depicts the variance in postmortem fentanyl concentrations among the 165 cases in which the parent drug was detected from 2013 through 2018.

The drugs most commonly used in conjunction with fentanyl or a fentanyl analog were benzodiazepines (74 of 197 cases; 38%), morphine (52 of 197 cases; 26%), heroin (41 of 197 cases; 21%), cocaine (34 of 197 cases; 17%), and marijuana (33 of 197 cases; 17%). Because morphine is a metabolite of heroin, the cases in which morphine was detected could actually be due to heroin use. Other drugs of abuse detected in <17% of cases were other opiates and methamphetamine.

### Demographics

Of all fentanyl-related overdoses, 78% of decedents were male (153 decedents), 22% female (44 decedents), 83% Caucasian (164 decedents), and 16% African American (31 decedents). The average age reported was 39 years (range, 22 to 69 years). The demographic most affected by fentanyl overdose in Jefferson Parish, LA was white males (63% of all deaths, 124 decedents).

## DISCUSSION

The data collected during this 6-year study verified that America's opioid epidemic had reached the southeast region of the United States. This study demonstrates that the number and proportion of fentanyl-related overdose deaths increased rapidly during the 6-year period, from 4% to 45% of all drug deaths. From 2013 through 2018, fentanyl-related opioid deaths increased approximately 1,200% in Jefferson Parish, LA. A slow increase during the first 3 years was followed by a near doubling of cases in 2016, a tripling of cases in 2017, and a 6-fold increase in incidence in 2018. The 78 deaths in 2018 account for 40% of the 197 total fentanyl-related overdoses from 2013 through 2018, while 2017 and 2018 overdoses combined (144 deaths) account for 73% of the fentanyl-related overdoses.

The types of fentanyl analogs used recreationally also sharply increased during the first 5 years of the study. One analog was reported in 2015, and 6 analog types were reported in 2017, indicating that the drug was being manipulated for recreational use. The reason for the sudden drop from 2017 (6 types) to 2018 (1 type) in fentanyl analogs detected is unknown. Drug synthesis outside of the regulated pharmaceutical market, which only produces the parent fentanyl, leads to modifications in the chemical structure that result in new derivatives of the parent drug. The analogs have essentially the same effect on the human body as the parent drug fentanyl.^3^ Recreational manipulation may be an attempt to increase efficacy and avoid detection by law enforcement. Manipulation may also result in analogs escaping medical laboratory detection.

The multiple drugs identified by GC/MS in almost 74% of fentanyl-related deaths in 2017 suggest that polysubstance abuse was common among the decedents. When one substance is laced with another, monitoring or predicting the potency of any particular dose is extremely difficult. Fentanyl and its analogs are rarely seen as independent substances. Determining the specific dose of fentanyl necessary to cause death is difficult because of the large variability between antemortem doses and postmortem blood concentrations.^12^ The lethal dose of fentanyl ranges from 3 to 200 ng/mL.^13^ As stated earlier, the average fentanyl concentration detected in this study was 17.62 ng/mL, which falls into the listed lethal dose range.

Future research into the characteristics of each death may help to create a better picture of what a deadly dose of fentanyl or a fentanyl analog may look like. As the number of fentanyl-related deaths continues to rise, in-depth analysis of the presence of other drugs at the time of death may help identify the mixtures that are most deadly in combination with fentanyl and its derivatives.

A limitation of this study was the inability to distinguish between prescription fentanyl and illicit fentanyl. The distinction is not made in the analyses because death investigators often cannot make that determination based on scene findings, and the toxicology analyses cannot differentiate between licit and illicit fentanyl, resulting in inconclusive information.

## CONCLUSION

Fentanyl-related deaths are an emerging and troublesome problem. The data from this study validate the hypothesis that the fentanyl epidemic has affected Jefferson Parish in southeast Louisiana. Identifying the means and implementing interventions to reduce the number of deaths attributable to fentanyl and other illicit drugs are major challenges at the community, state, and national levels. Efforts must be targeted toward overdose prevention among drug users, recovery programs, and naloxone (fentanyl overdose antagonist) availability. Educating young school-age children who have not been exposed to the world of illicit drug use is also imperative to help counter this trend.

## Notes

This article meets the Accreditation Council for Graduate Medical Education and the American Board of Medical Specialties Maintenance of Certification competencies for Patient Care and Medical Knowledge.
